# Association Between HLA‐DRB1 Serotype and HLA‐DQB1 Allele Mismatches and Acute Rejection in Kidney Transplantation

**DOI:** 10.1111/tan.70228

**Published:** 2025-05-22

**Authors:** Renato de Marco, Isau H. Noronha, Luiza Zainotti Miguel Fahur Bottino, Tuila Bittencourt Mourão, Gisele Fabianne Rampim, João Campos, Alberto Cardoso Martins Lima, Lúcio Requião‐Moura, Hélio Tedesco‐Silva, José Medina Pestana, Maria Gerbase‐DeLima

**Affiliations:** ^1^ Instituto de Imunogenética (IGEN) Associação Fundo de Incentivo à Pesquisa (AFIP) São Paulo São Paulo Brazil; ^2^ Nephrology Division Universidade Federal de São Paulo São Paulo São Paulo Brazil; ^3^ Hospital do Rim Fundação Oswaldo Ramos São Paulo São Paulo Brazil

**Keywords:** acute rejection, amino acid mismatches, EMMA, Eplet, HLA compatibility, HLA mismatch, HLA serotypes, molecular mismatches, PIRCHE

## Abstract

The purpose of this single‐center case–control study was to investigate the association between HLA serotype mismatch (MM), compared to other HLA MM modalities, and the occurrence of acute rejection (AR) within the first year after deceased donor kidney transplantation. The study included 198 transplants in 99 pairs of recipients of kidneys from the same donor, where one recipient experienced AR and the other survived the first year without AR. Donors and recipients were typed with NGS for 11 HLA loci at high resolution. HLA MM categories included allele groups, alleles, serotypes, amino acids, EMMA, eplet and PIRCHE‐II. Additionally, we investigated Cytomegalovirus LIL peptide (CMV LIL) MM. Recipients with AR presented higher frequencies of pre‐transplant HLA‐ABDR DSA (20.2% vs. 6.1%, *p* = 0.005) and CMV LIL MM (24.2% vs. 10.1%, *p* = 0.01). Univariate and multivariate Cox proportional hazards regression for matched‐pair analyses were used to test the association between HLA MM and AR. Univariate analyses indicated significant association with DRB1 ST, HLA‐DQB1 AG, HLA‐DQB1 AL, EMMA C, EMMA DQB1, Eplet ABC and Eplet DQ MM. Different models were tested in multivariate analyses, all including pre‐transplant HLA‐ABDR DSA and CMV LIL MM. The models were compared using the Akaike Information Criterion (AIC). The best estimate for AR prediction (AIC = 97.6) was the model that included pre‐transplant HLA‐ABDR DSA (HR = 11.97; *p* = 0.003), CMV LIL MM (HR = 367.2; *p* < 0.001), HLA‐DRB1 serotype MM (9.65; *p* = 0.002) and HLA‐DQB1 allele MM (HR = 3.54; *p* = 0.033). In conclusion, this original report demonstrates an association between the HLA‐DRB1 serotype MM and AR, highlighting that serotypes are clinically relevant.

**Abbreviations**
AA: Amino acids
AA‐MM: Amino acid mismatch
AG‐MM: Antigen group mismatch
AIC: Akaike Information Criterion
Al‐MM: Allele mismatch
AR: Acute rejection
AZA: Azathioprine
CI: Confidence interval
CIT: Cold ischemia time
CIWD: Common, intermediate and well‐documented
CMV: Cytomegalovirus
CNI: Calcineurin inhibitor
cPRA: Calculated class I and II panel reactive antibodies
DD: Deceased donor
DEP: Determining epitopes
DSA: Donor‐specific antibody
DSA‐ABDR: HLA‐A, ‐B, ‐DR donor‐specific antibodies
DSA‐CDQDP: HLA‐C, ‐DQ, ‐DP donor‐specific antibodies
EMMA: Epitope Mismatch Algorithm
HLA: Human Leukocyte Antigen
HR: Hazard ratio
IQR: Interquartile range
MFI: Mean fluorescence intensity
MM: Mismatch
mMM: Molecular mismatch
NGS: Next‐generation sequencing
PIRCHE: Predicted Indirectly Recognisable HLA Epitopes
rATG: Rabbit anti‐thymocyte globulin
rSSO: Reverse sequence‐specific oligonucleotide
SAB: Luminex single antigen bead assay
SD: Standard deviation
ST: Serotype
ST‐MM: Serotype mismatch
TCMR: T cell‐mediated rejection
WHO: World Health Organisation

## 1 Introduction

Association between serologically defined HLA‐A, ‐B, ‐DR mismatches (MM) and adverse outcomes in deceased donor (DD) kidney transplantation has been observed in several unicentric and multicentric studies [1, 2, 3, 4, 5, 6]. More recently, it has been shown that HLA‐DQB1 and HLA‐DQA1 MM also impact graft outcomes [7, 8, 9, 10], whereas no association has been observed with HLA‐DP antigen or allele MM [11, 12, 13].

HLA‐A, ‐B, ‐DR compatibility has been successfully used as a major criterion in DD kidney allocation algorithms since the 1970s. However, as immunosuppression regimens have become more efficient in the past two decades, the impact of HLA‐A, ‐B, ‐DR MM on graft outcomes has decreased to a point that is being questioned [14, 15]. For example, in the United States, where issues related to racial disparity in access to transplantation also question this policy, a stepwise reduction in the weight of HLA‐A, ‐B, ‐DR MM in the allocation algorithm has already been implemented [7, 16].

Nevertheless, while the impact on transplant outcomes of HLA compatibility defined at antigenic level is declining, several new approaches have been proposed to refine HLA compatibility beyond antigenic level. Thus, careful analysis of these new HLA compatibility modalities should be pursued before altogether discarding HLA matching from DD organ allocation algorithms.

The advent of DNA‐based HLA typing methods has led to the description of thousands of HLA alleles, and over 39,000 classical class I and class II alleles have been assigned to the IPD‐IMGT/HLA Database [17], as of September 2024. The latest publication on the equivalence between alleles and serologic specificities, based on the reactivity of sera and monoclonal antibodies on high resolution HLA typed cells or solid phase assays with single antigen beads, contains information on 1,262 class I and 336 class II HLA alleles [18]. Therefore, many HLA alleles still lack serological equivalence, which poses a problem for antibody assessment. While the Luminex single antigen bead assay (SAB) is considered the gold standard for antibody identification, it does not enable testing the serum against all.

Recently, a new in silico strategy has been proposed for systematically classifying all HLA alleles into serological specificities, termed serotypes (ST). This algorithm is based on amino acid (AA)‐determining epitopes (DEP) on class I (20–25 DEPs) and class II (13 DEPs) HLA molecules. It allows the definition of all the 82 class I and the 35 class II World Health Organisation (WHO) recognised HLA‐specificities [17] and, in addition, 85 novel HLA class I and 21 novel HLA‐DRB1 serologic specificities corresponding to common alleles [19, 20].

The knowledge of the AA sequences and the tertiary structure of class I and class II HLA molecules has led to the proposal of different types of molecular MM (mMM) for HLA incompatibility assessment. The two modalities of mMM that have been mostly investigated are the Eplet MM (HLAMatchmaker) [21, 22] and the Predicted Indirectly Recognisable HLA Epitopes (PIRCHE‐II) algorithm [23]. Other mMM modalities consider the total number of mismatched AA (AA‐MM) or selected AA MM regarding AA physicochemical properties [24], or solvent‐accessible AA, as the Epitope Mismatch Algorithm (EMMA) [25].

Several studies have investigated the association of the different types of mMM, alone or in combination, with poor kidney graft outcomes, such as de novo HLA‐donor‐specific antibodies (DSA), rejection and graft loss [23, 24, 25, 26, 27, 28, 29, 30, 31, 32, 33, 34, 35, 36, 37, 38, 39, 40, 41, 42, 43, 44, 45, 46, 47]. Despite some promising results shown in some of these studies, it is generally agreed that further studies regarding the refinement of mMM analyses, as well as regarding quantitative mMM issues, are necessary before considering one or a combination of mMM types for clinical decision making and/or for DD kidney allocation [48, 49].

The primary interest of the current study was to evaluate the impact of HLA‐ST MM [20], as compared to antigen group MM (AG‐MM) allele MM (AL‐MM), on the occurrence of acute rejection (AR) within the first year after DD kidney transplantation. In addition, we investigated the impact of HLA mMM, including AA, EMMA, Eplet and PIRCHE modalities. We included CMV LIL peptide MM (CMV LIL MM) as a covariate because a recent study showed that CMV seropositive recipients receiving a kidney from a donor with certain HLA‐A or C MM are at a higher risk for T cell mediated rejection (TCMR). This association is explained by the fact that some HLA‐A or ‐C alleles share AA sequences with peptides produced by specific CMV strains. Characteristically, these rejections occur within the first 90 days, and particularly within the first 2 weeks after transplantation. The original publication included a validation cohort containing all the biopsy‐proven TCMR of our current study that showed an association of CMV‐LIL peptide MM with rejection [50].

## 2 Materials and Methods

### 2.1 Patient Population

This retrospective, single‐center, case–control study included 198 first mate‐kidney transplant recipients, of whom one presented AR within the first year after transplantation and the other survived the first year without AR. The transplants were performed between 2014 and 2019 at Hospital do Rim, Fundação Oswaldo Ramos, São Paulo–Brazil. Exclusion criteria included donor‐recipient age mismatch (age < 18 or > 18 years) and lack of stored donor or recipient biological material for high‐resolution HLA typing in our laboratory. CMV positivity was present in 92% of the patients, and none received CMV pharmacological prophylaxis.

The transplants were performed with a pre‐transplant negative T and B complement‐dependent cytotoxicity crossmatch and no HLA‐A, ‐B or ‐DR DSA in current or historical sera, with mean fluorescence intensity (MFI) > 1,500 in the SAB assay (One Lambda, Thermo Fisher Scientific, Canoga Park, CA), as previously described [51].

The maintenance immunosuppressive regimens consisted of calcineurin inhibitors and prednisone combined with a third drug chosen based on calculated panel reactive antibodies (cPRA) or donor profile: mycophenolic acid for recipients with cPRA > 50% or those receiving extended criteria donors, and azathioprine for cPRA ≤ 50% recipients or those receiving standard criteria donors.

The covariates of interest were obtained from clinical files. They included characteristics from the donor (age and sex), the recipient (age at transplantation, sex, race, % calculated panel reactive class I and class II antibodies (cPRA), immunosuppression), and from the transplant (cold ischemia time, HLA‐A, B, DR antigen mismatches).

The study was approved by the local ethics committee of the Federal University of São Paulo (approval number: 6838623.9.0000.550).

### 2.2 Study Endpoint

All treated rejections that occurred within the first year after transplantation were included in the analysis. No recipient presented more than one AR episode. The 90 biopsy‐proven ARs were classified according to the 2007 Banff criteria [52] comprised 88 TCMR (22, IA, 7, IB, 12, IIA, 6, IIB, 1, III, 40, borderline) and two mixed ARs. Clinically diagnosed rejections (*n* = 9) were defined when recipients presented acute impairment in the graft function, were treated as rejections without a biopsy, and the creatinine decreased after the treatment.

### 2.3 HLA Genotyping

Kidney recipients and donors were retrospectively typed for HLA ‐A, ‐B, ‐C, ‐DRB1, DRB345, ‐DQA1, ‐DQB1, ‐DPA1 and ‐DPB1 loci at high resolution, using next‐generation sequencing (NGS) on an Illumina platform (Illumina, San Diego, CA, USA) with the kit Fastplex (One Lambda).

### 2.4 HLA Mismatches Determination

HLA antigen MM were defined considering allele groups, except for HLA‐DPB1, for which a correspondence between antigens and allele groups does not apply [53]. Allele MM were defined at the P‐group level. Serotype MM were classified according to Osoegawa et al. [20], using a locally developed online tool for obtaining the correspondence between alleles and serotypes, available at https://sorotipos18ws‐v2.igen.org.br/en‐US. Amino acid MM corresponded to the number AA present in the donor and absent in the recipient. HLA EMMA class I intra‐locus and HLA‐DRB1/3/4/5, DQA1/B1, DPA1/B1 inter‐loci MM were defined using the EMMA software version 1.06. Using a local developer program, Eplet MM load was determined based on the algorithm available at https://www.epregistry.com.br version 2024–08. PIRCHE‐II scores were calculated using the algorithm available at https://www.pirche.com. CMV‐LIL MM were corresponded to HLA‐A or ‐C MM [50].

### 2.5 Statistical Methods

Baseline univariate analyses were conducted to determine adjustments for subsequent regression models. Continuous variables were assessed for distributional assumptions using the Shapiro–Wilk test. Variables following a normal distribution were analysed using Student's *T*‐test, whereas non‐normally distributed variables were analysed with the Mann–Whitney U test. Categorical variables were compared using Fisher's exact test.

Univariable Cox proportional hazards regression models were then applied to evaluate the association of individual covariates with rejection‐free survival. The matched‐pair nature of the data, reflecting donor‐recipient pairs, was accounted for by stratification on pairwise matching. This approach ensured robust estimates while respecting the inherent pairing structure of the data. The proportional hazards assumption was assessed for all covariates included in the univariable Cox models using Schoenfeld residuals.

Covariates with *p* values ≤ 0.05 in the univariable analysis were selected for inclusion in multivariable models. In cases where variables were found to be correlated, the variable with the higher hazard ratio (HR) was retained for the final models to minimise redundancy.

Multiple multivariable models were constructed and compared using the Akaike Information Criterion (AIC) to identify the optimal model. The model with the lowest AIC value, indicating the best balance between goodness‐of‐fit and parsimony, was selected as the final model.

Statistical significance was defined as *p* < 0.05. All analyses were performed using Stata 18.0.

## 3 Results

### 3.1 Baseline Characteristics

The baseline characteristics of the transplants with and without AR are shown in Table 1. Results of numerical variables are presented as mean and standard deviation (SD), or as median and inter‐quartile range (IQR) for variables with or without normal distribution, respectively. Recipient and transplant characteristics did not significantly differ between the groups, except for pre‐transplant HLA‐A, ‐B, ‐DR DSA < 1500 MFI and CMV LIL MM. As donor variables were identical, the study was performed on mate‐kidney recipients. Maintenance immunosuppressive regimens consisted of a calcineurin inhibitor (> 98% tacrolimus) in combination with azathioprine (37.4%), mycophenolate sodium (51.5%) or another drug (11.1%).

**TABLE 1:** Baseline characteristics of transplants with and without rejection within the first year after transplantation.

Variables	Rejection		*p*
	Yes, *n* = 99	No, *n* = 99	
Recipient age, years, median (IQR)	43 (4.0–70.0)	46 (4.0–79.0)	0.644
Recipient age‐goups			0.903
0–17, *n* (%)	10 (10.1)	10 (10.1)	
18–49, *n* (%)	51 (51.5)	48 (48.5)	
≥ 50, *n* (%)	38 (38.4)	41 (41.4)	
Recipient sex, male, *n* (%)	67 (67.7)	63 (63.6)	0.654
Recipient race			0.473
Caucasian, *n* (%)	45 (45.5)	40 (40.4)	
Non Caucasian, *n* (%)	54 (54.5)	68 (68.6)	
Class I + II cPRA > 0%, *n* (%)	27 (27.3)	20 (20.2)	0.244
DSA‐ABDR (MFI < 1500), *n* (%)	20 (20.2)	6 (6.1)*	**0.005**
DSA‐CDQDP (MFI > 300) *n* (%)	16 (16.2)	9 (9.1)	0.199
CIT, hours, mean (SD)	23.3 (0.80)	23.5 (0.74)	0.796
DGF, *n* (%)	56 (56.7)	53 (53.5)	0.775
Prolonged (> 14 days) DGF, *n* (%)	11 (11.1)	10 (10.1)	0.818
Immunosuppression			0.625
CNI + AZA, *n* (%)	37 (37.4)	36 (36.4)	
CNI + MPS, *n* (%)	51 (51.5)	54 (54.5)	
Other, *n* (%)	11 (11.1)	9 (9.1)	
CMV LIL peptide MM, *n* (%)	24 (24.2)	10 (10.1)	**0.010**
Donor age years, median (IQR)	49 (37.0–56.0)		—
Donor sex, male, *n* (%)	114 (57.6)		—

*Note:* Significant *p* value is in bold.

Abbreviations: AZA, azathioprine; CIT, cold ischemia time; CMV, Cytomegalovirus; CNI, calcineurin inhibitor; cPRA, calculated panel reactive antibodies; DGF, delayed graft function; DSA‐ABDR, HLA‐A, ‐B, ‐DR donor‐specific antibodies; DSA‐CDQDP, HLA‐C, ‐DQ, ‐DP donor‐specific antibodies; MFI, mean fluorescence intensity; MM, mismatch; MPS, mycophenolate sodiun.

* One recipient was inadvertently transplanted with an HLA‐B13 DSA with 8000 MFI.

Donors median age was 48 years (8–72) and 60% were males.

Pre‐transplant HLA‐A, ‐B, ‐DR DSA < 1500 MFI and CMV LIL MM were also subjected to the univariate Cox proportional regression for matched‐pair analysis that showed that the association of CMV LIL MM and rejection violated the Cox proportional hazards assumption. Therefore, CMV LIL MM was entered as a time‐varying covariate in the multivariate models.

### 3.2 Univariate Analyses of HLA MM and Rejection

Associations between HLA AG, AL and ST MM and rejection are presented in Table 2. The numbers of different HLA‐DRB1 serotypes and alleles, corresponding to each allele group, identified in the recipients and the donors from the present study are shown in Table S1. HLA DRB1 ST, HLA‐DQB1 AG and HLA‐DQB1 AL MM were significantly associated with rejection. Regarding the associations with HLA‐DQB1, the AL MM was chosen to be included in the multivariate analyses considering its higher HR.

**TABLE 2:** Comparison of HLA allele group, allele, and serotype mismatches (MM) between transplants with and without rejection. Univariate stratified Cox proportional hazard regression for matched‐pair analysis.

1 + 2 vs. 0 MM comparisons	Allele group mismatches			Allele mismatches			Serotype mismatches		
	HR	95% CI	*p*	HR	95% CI	*p*	HR	95% CI	*p*
HLA‐A	1.00	0.42–2.40	1.000	2.67	0.71–10.05	0.147	1.38	0.55–3.42	0.493
HLA‐B	2.00	0.60–6.64	0.258	2.00	0.50–8.00	0.327	1.67	0.40–6.98	0.484
HLA‐C	2.67	0.71–10.05	0.147	1.20	0.37–3.93	0.763	1.40	0.44–4.41	0.566
HLA‐DRB1	2.67	0.71–10.05	0.147	1.57	0.61–4.05	0.350	**2.71**	**1.14–6.46**	**0.024**
HLA‐DQB1	**2.22**	**1.01–4.88**	**0.047**	**2.38**	**1.04–5.43**	**0.040**	2.25	0.98–5.17	0.056
HLA‐DQA1	1.33	0.63–2.82	0.451	1.80	0.83–3.90	0.136	1.33	0.63–2.81	0.451
HLA‐DPB1*	—	—	—	1.00	0.29–3.45	1.000	1.00	0.29–3.45	1.000
HLA‐DPA1	0.70	0.35–1.39	0.306	0.74	0.37–1.47	0.386	0.88	0.44–1.77	0.724
HLA‐DRB345	1.67	0.61–4.59	0.323	1.00	0.46–2.16	1.000	1.00	0.46–2.16	1.000

*Note:* Significant *p* value is in bold.

Abbreviations: CI, confidence interval; HR, hazard ratio.

* The classification in allele groups does not apply for HLA‐DPB1.

Associations between AA‐MM and EMMA‐MM and rejection are described in Table 3. HLA‐C AA and EMMA MM, HLA‐DQA1/B1 AA and EMMA MM, HLA‐DQB1 AA and EMMA MM were significantly associated with rejection. The EMMA MM was chosen to be included in the multivariate analyses considering its higher HR.

**TABLE 3:** Comparison of amino acid (AA) mismatches (MM) and Epitope Mismatch Algorithm (EMMA) MM between cases with and without rejection. Univariate analyses using stratified Cox proportional hazard regression for matched‐pair analysis.

Mismatches	AA MM					EMMA				
	Rejection (*N* = 99)	No rejection (*N* = 99)	HR	95% CI	*p*	Rejection (*N* = 99)	No rejection (*N* = 99)	HR	95% CI	*p*
HLA‐A, median (IQI)	13 (6–21)	12 (7–19)	1.00	0.97–1.04	0.798	10 (3–16)	9 (3–14)	1.01	0.96–1.05	0.821
HLA‐B, median (IQI)	12 (6–18)	12 (6–17)	1.02	0.97–1.07	0.451	6 (3–10)	6 (2–9)	1.02	0.94–1.11	0.638
HLA‐C, median (IQI)	12 (7–16)	9 (4–14)	**1.06**	**1.01–1.12**	**0.029**	7 (3–9)	5 (2–8)	**1.09**	**1.01–1.19**	**0.034**
HLA‐ABC, median (IQI)	39 (24–49)	35 (25–45)	1.02	0.99–1.04	0.125	23 (15–30)	21 (12–28)	1.02	0.99–1.06	0.196
HLA‐DRB1/3/4/5, median (IQI)	3 (1–7)	2 (0–6)	1.03	0.99–1.09	0.164	2 (1–5)	2 (0–4)	1.04	0.98–1.12	0.187
HLA‐DQA1/B1, median (IQI)	9 (0–31)	2 (0–18)	**1.02**	**1.00–1.05**	**0.040**	6 (0–22)	2 (0–13)	**1.03**	**1.00–1.06**	**0.034**
HLA DQA1, median (IQI)	1 (0–14)	0 (0–8)	1.04	0.99–1.08	0.088	1 (0–11)	0 (0–7)	1.05	0.99–1.10	0.089
HLA DQB1, median (IQI)	4 (0–17)	1 (0–11)	**1.05**	**1.01–1.09**	**0.029**	3 (0–14)	1 (0–8)	**1.06**	**1.01–1.12**	**0.018**
HLA‐DPA1/B1, median (IQI)	8 (2–16)	7 (3–18)	0.98	0.94–1.03	0.513	6 (1–11)	5 (1–14)	0.98	0.92–1.04	0.466
HLA DPA1, median (IQI)	0 (0–6)	0 (0–6)	0.98	0.87–1.10	0.705	0 (0–6)	0 (0–6)	0.98	0.87–1.10	0.719
HLA DPB1, median (IQI)	6 (2–11)	6 (3–12)	0.98	0.91–1.05	0.505	3 (1–8)	3 (1–9)	0.96	0.87–1.06	0.407

*Note:* Significant *p* value is in bold.

Abbreviations: CI, confidence interval; HR, hazard ratio.

Associations between Eplet MM Load and rejection are described in Table 4. HLA‐ABC and HLA‐DQA1/B1 MM load were significantly associated with rejection, and HLA‐DQA1/B1 MM load was chosen to be included in the multivariate analyses, considering its higher HR.

**TABLE 4:** Comparison of Eplet load between cases with and without rejection. Univariate analyses using the stratified Cox proportional hazard regression for matched‐pair analysis.

Eplet MM load	Rejection (*N* = 99)	No rejection (*N* = 99)	HR	95% CI	*p*
HLA‐ABC, median (IQI)	26 (18–34)	24 (16–32)	**1.04**	**1.00–1.08**	**0.046**
HLA‐DRB1/3/4/5, median (IQI)	3 (1–6)	2 (0–6)	1.05	0.99–1.11	0.125
HLA‐DQA1/B1, median (IQI)	6 (0–11)	1 (0–9)	**1.08**	**1.01–1.15**	**0.019**
HLA‐DPA1/B1, median (IQI)	9 (4–13)	9 (4–15)	0.99	0.93–1.06	0.768

*Note:* Significant *p* value is in bold.

Abbreviation: IQI, interquartile interval.

No association with rejection was observed concerning PIRCHE‐II MM (Table 5).

**TABLE 5:** Comparison of PIRCHE scores between cases with and without reaction. Univariate analyses using stratified Cox proportional hazard regression for matched‐pair analysis.

PIRCHE‐II scores	Rejection (*N* = 99)	No rejection (*N* = 99)	HR	95% CI	*p*
HLA‐ABCDRDQDP, median (IQI)	336 (235–452)	300 (214–455)	1.00	0.99–1.00	0.144
HLA‐ABCDRB1DQ1, median (IQI)	59 (37–85)	50 (32–80)	1.01	0.99–1.02	0.255

Abbreviations: CI, confidence interval; HR, hazard ratio.

All the variables included in the univariate analyses between HLA MM and rejection adhere to the Cox proportional hazards regression assumption.

### 3.3 Multivariate Analyses of HLA MM and Rejection

Associations between HLA mismatches and AR, adjusted for pre‐transplant HLA‐A, B, DR donor‐specific antibodies and CMV LIL mismatches are presented in Table 6.

**TABLE 6:** Association between HLA mismatches (1 + 2 vs. 0) and rejection, adjusted for pre‐transplant HLA‐A, B, DR donor‐specific antibodies and CMV LIL mismatch as a time‐varying variable, in different models including only non‐collinear variables. Analyses using the stratified Cox proportional hazard regression for matched‐pair method.

Models	AIC	Variables	HR	95% CI	*p*
Model 1 (no covariates)	114.4	DSA‐ABDR	5.81	1.48–22.71	0.011
		CMVLIL MM	68.4	3.31–1412	0.006
Time dependent variable		CMV LIL MM (time dep.)	0.98	0.95–0.99	0.014
Model 2 (best model)	97.6	DSA‐ABDR	11.97	2.33–61.49	0.003
		CMV LIL MM	367.2	12.77–10558	0.001
		HLA‐DQB1 AL MM	3.54	1.11–11.28	0.033
		HLA‐DRB1 ST MM	9.65	2.32–40.06	0.002
Time dependent variable		CMV LIL MM (time dep.)	0.97	0.95–0.99	0.005
Model 3	100.9	DSA‐ABDR	11.64	2.34–57.98	0.003
		CMV LIL MM	165.5	7.48–3659	0.001
		HLA‐DRB1 ST MM	9.05	2.32–35.24	0.002
Time dependent variable		CMV_LIL MM (time dep.)	0.97	0.96–0.99	0.006
Model 4	110.4	DSA‐ABDR	5.99	1.44–24.91	0.014
		CMV LIL MM	84.46	4.26–1673	0.004
		HLA‐DQB1 AL MM	3.31	1.18–9.27	0.023
Time dependent variable		CMV_LIL MM (time dep.)	0.98	0.96–0.99	0.014
Model 5	110.3	DSA‐ABDR	4.11	1.00–16.94	0.050
		CMV LIL MM	77.33	3.54–1688	0.006
		EMMA C (IQI: 2–9)	1.10	0.98–1.22	0.102
		EMMA DQB1 (IQI: 0–14)	1.07	1.01–1.14	0.021
Time dependent variable		CMV_LIL MM (time dep.)	0.97	0.95–0.99	0.005
Model 6	111.0	DSA‐ABDR	5.55	1.30–23.73	0.021
		CMV LIL MM	72.14	3.37–1544	0.006
		Eplet ABC (IQI: 16–34)	1.03	0.99–1.07	0.225
		Eplet DQ (IQI: 0–11)	1.07	0.99–1.16	0.090
Time dependent variable		CMV_LIL MM (time dep.)	0.98	0.96–0.99	0.017
Model 7	111.13	DSA‐ABDR	4.88	1.22–19.57	0.025
		CMV LIL MM	68.63	3.41–1380	0.006
		EMMA DQB1 (IQI: 0–14)	1.07	1.01–1.13	0.031
Time dependent variable		CMV_LIL MM (time dep.)	0.98	0.96–0.99	0.018
Model 8 (combined)	98.67	DSA‐ABDR	11.16	2.14–58.13	0.004
		CMV LIL MM	368.3	13.16–10310	0.001
		HLA‐DQB1 AL	2.82	0.80–9.98	0.108
		HLA‐DRB1 ST MM	9.45	2.25–39.62	0.002
		EMMA DQB1 (IQI: 0–14)	1.03	0.97–1.11	0.344
Time dependent variable		CMV_LIL MM (time dep.)	0.97	0.95–0.99	0.005

Abbreviations: AL, allele; CI, confidence interval; CMV, Cytomegalovirus; DSA‐ABDR, HLA‐A, ‐B, ‐DR donor‐specific antibodies; EMMA, Epitope Mismatch Algorithm; HR, hazard ratio; IQR, Interquartile range; MM, mismatch; ST, serotype.

CMV LIL MM was entered as a time‐varying covariate in multivariate analyses with HLA MM because it violated the Cox proportional hazards regression assumption. Thus, the results of the Cox regression considering CMV LIL MM as a time‐dependent variable are also described.

Different models were tested in multivariate analyses in order to avoid collinearity among the variables.

The model including DSA‐ABDR, CMV LIL MM, DQB1 AL and DRB1 ST (model # 2) was chosen as the optimal model because it presented the lowest AIC value, indicating the best balance between goodness‐of‐fit and parsimony. The HR for HLA‐DRB1 ST MM was higher (9.65) than the HR for HLA‐DQB1 AL MM [53].

## 4 Discussion

This single‐center case–control study was designed to examine the association between HLA serotypes MM and AR within the first year after kidney transplantation. In addition, we investigated HLA incompatibilities, defined in terms of allele groups, alleles, AA, EMMA, Elet and PIRCHE MM.

The original and most relevant finding of this investigation was the independent and strong association of HLA‐DRB1 ST MM with AR, whereas no association was detected with HLA‐DRB1 AG or AL MM. Furthermore, weaker or no associations were observed with EMMA, Eplet or PIRCHE‐II molecular MM.

The association of HLA‐DRB1 serotype MM, but not with allele group or allele MM, shows that serotype MM is enough to gain the benefit of HLA‐DRB1 match in kidney transplantation. Among the 13 HLA‐DRB1 AG and the 40 serotypes presently recognised, all the AG and 26 serotypes were identified in at least one recipient or donor of our study. As expected, the proportion of transplants performed with zero HLA‐DRB1 serotype MM was lower (42.4%) than that with zero AG MM (76.3%).

In addition to HLA‐DRB1 ST MM, HLA‐DQB1 AL MM, pre‐transplant, HLA‐A, − B or ‐DR DSA with MFIs below 1500, and CMV LIL peptide MM were also associated with AR. The association of DQB1 AL‐MM with AR aligns with previous publications on the relationship between HLA‐DQB1 and ‐DQA1 AL MM and poor graft outcomes [7, 8, 9, 10]. However, the impact of DRB1 ST MM was stronger than that of DQB1 AL‐MM. In the different multivariant analysis models, the HR for HLA‐DRB1 ST MM and HLA‐DQB1 AL MM ranged between 9.0–9.6 and 2.8–3.5, respectively.

The association between weak (300 > MFI < 1,500) pre‐transplant HLA‐A, B, DR DSA and AR was unexpected, considering that a previous study from our center showed that only HLA‐A, ‐B, ‐DR DSA with MFIs > 1,500 impacted on graft survival [54]. Consequently, since 2009, we have routinely avoided transplants in the presence of HLA‐A, ‐B, ‐DR DSA with MFI > 1500 [51]. The risk for AR conferred by weak HLA‐A, ‐B, ‐DR DSA should not, in our opinion, be considered a contra‐indication for transplantation, considering the current low rates of AR, their successful treatment, and the lack of evidence that these DSA would affect graft survival.

The association of CMV‐LIL‐MM with rejection was expected, considering the previous study [50], described in the Introduction.

The strength of this study includes its case–control design, the high‐resolution HLA typing of all recipients and donors, and the evaluation of other HLA compatibility modalities in addition to matching at antigen, allele and serotype levels. On the other hand, there are limitations. It is a single‐centre study in low‐risk transplants, with 76.3% of the transplants performed with zero HLA‐DRB1 AG‐MM and, therefore, our results might not be directly applicable to other populations. Additionally, considering the number of transplants analysed in relation to the multiple tested variables, we adopted a conservative approach in the statistical analysis. Consequently, some associations of HLA MM and AR might not have been adequately considered.

Further studies of the impact of HLA‐DRB1 ST‐MM, isolated or in combination with HLA‐DQB1 AL MM, on other kidney transplant outcomes, including *de novo* DSA and long‐term graft survival, are urgently needed because matching for HLA‐DRB1 ST represents a very promising tool for assessing transplant immunologic risk and for inclusion in DD kidney allocation algorithms. Although reliable serotype determination depends on accurate HLA alleles identification, high‐resolution HLA typing of DD at the time of transplantation is not a sine qua non condition, since reverse sequence‐specific oligonucleotide (rSSO) typing kits that define common, intermediate and well‐documented (CIWD) alleles are usually sufficient for correct serotypes assignment. It should be noted, however, that advances in nanopore sequencing technology have allowed a high‐resolution HLA typing within a short turnaround time. In fact, we have been successfully using nanopore sequencing for 11 HLA loci high‐resolution typing for DD since October 2024.

In conclusion, this study demonstrated, for the first time, the independent association between HLA‐DRB1 ST‐MM and AR in kidney transplantation, showing that the newly proposed HLA serotype classification is not only an excellent approach for HLA antibody analyses [19, 20], but is also clinically pertinent.

## Author Contributions

R.d.M. participated in the study design, performance of the research, data curation and analysis, and in the writing of the manuscript; I.H.N., L.Z.M.F.B., T.B.M., G.F.R. and J.C. participated in the performance of the research; A.C.M.L. participated in the data analysis and in the revision of the manuscript; L.R.M. participated in the data curation, data analysis and in the revision of the manuscript; J.M.P. and H.T.S. participated in the revision of the manuscript; M.G.‐D. participated in the study design, data analysis and in the writing of the manuscript. All authors read and approved the manuscript.

## Conflicts of Interest

The authors declare no conflicts of interest.

## Supporting information


Table S1.

## Data Availability

The data that support the findings of this study are available on request from the corresponding author. The data are not publicly available due to privacy or ethical restrictions.
