# Supplementary material for: Association Between HLA‐DRB1 Serotype and HLA‐DQB1 Allele Mismatches and Acute Rejection in Kidney Transplantation
Source: HLA. 2025 May 22;105(5):e70228. doi: 10.1111/tan.70228 (PMC12096148; doi:10.1111/tan.70228)
Supplement: Supplementary file 1 — Table S1. [file TAN-105-e70228-s001.docx]

**Supplementary Table** **1**. Numbers of different HLA-DRB1 serotypes and alleles, corresponding to each allele group, identified in the recipients and the donors from the present study.

| **Allele Groups** | **Recipients** | | **Donors** | |
| --- | --- | --- | --- | --- |
|  | **Allele (n)** | **Serotype (n)** | **Allele (n)** | **Serotype (n)** |
| DRB1*01 | DRB1*01:01 (14) | DR-0101 (30) | DRB1*01:01 (22) | DR-0101 (30) |
|  | DRB1*01:02 (16) |  | DRB1*01:02 (8) |  |
|  | DRB1*01:03 (1) | DR103 (1) | DRB1*01:03 (4) | DR103 (4) |
| DRB1*03 | DRB1*03:01 (31) | DR17 (31) | DRB1*03:01 (26) | DR17 (26) |
|  | DRB1*03:02 (6) | DR18 (6) | DRB1*03:02 (8) | DR18 (8) |
| DRB1*04 | DRB1*04:01 (9) | DR-0401 (41) | DRB1*04:01 (6) | DR-0401 (28) |
|  | DRB1*04:04 (18) |  | DRB1*04:04 (14) |  |
|  | DRB1*04:05 (13) |  | DRB1*04:05 (8) |  |
|  | DRB1*04:10 (1) |  |  |  |
|  | DRB1*04:02 (2) | DR-0402 (2) | DRB1*04:02 (6) | DR-0402 (6) |
|  | DRB1*04:03 (2) | DR-0403 (17) | DRB1*04:03 (8) | DR-0403 (20) |
|  | DRB1*04:06 (1) |  |  |  |
|  | DRB1*04:07 (7) |  | DRB1*04:07 (8) |  |
|  | DRB1*04:11 (7) |  | DRB1*04:11 (4) |  |
| DRB1*07 | DRB1*07:01 (39) | DR7 (39) | DRB1*07:01 (38) | DR7 (38) |
| DRB1*08 | DRB1*08:01 (8) | DR-0801 (30) | DRB1*08:01 (16) | DR-0801 (28) |
|  | DRB1*08:02 (5) |  | DRB1*08:02 (10) |  |
|  | DRB1*08:04 (8) |  | DRB1*08:04 (2) |  |
|  | DRB1*08:06 (1) |  |  |  |
|  | DRB1*08:07 (8) |  |  |  |
|  | DRB1*08:03 (2) | DR-0803 (2) | DRB1*08:03 (2) | DR-0803 (2) |
| DRB1*09 | DRB1*09:01 (13) | DR9 (13) | DRB1*09:01 (14) | DR9 (14) |
| DRB1*10 | DRB1*10:01 (13) | DR10 (13) | DRB1*10:01 (16) | DR10 (16) |
| DRB1*11 | DRB1*11:01 (30) | DR-1101 (45) | DRB1*11:01 (30) | DR-1101 (36) |
|  | DRB1*11:04 (14) |  | DRB1*11:04 (6) |  |
|  | DRB1*11:18 (1) |  |  |  |
|  | DRB1*11:02 (8) | DR-1102 (8) | DRB1*11:02 (14) | DR-1102 (14) |
|  |  |  | DRB1*11:03 (8) | DR-1103 (8) |
| DRB1*12 | DRB1*12:01 (9) | DR-1201 (9) | DRB1*12:01 (12) | DR-1201 (12) |
| DRB1*13 | DRB1*13:01 (20) | DR-1301 (44) | DRB1*13:01 (12) | DR-1301 (40) |
|  | DRB1*13:02 (24) |  | DRB1*13:02 (26) |  |
|  |  |  | DRB1*13:04 (2) |  |
|  | DRB1*13:03 (7) | DR-1303 (7) | DRB1*13:03 (6) | DR-1303 (6) |
|  | DRB1*13:05 (1) | DR-1305 (1) | DRB1*13:05 (2) | DR-1305 (2) |
| DRB1*14 | DRB1*14:01 (2) | DR-1401 (7) |  | DR-1401 (4) |
|  | DRB1*14:54 (5) |  | DRB1*14:54 (4) |  |
|  | DRB1*14:02 (2) | DR-1402 (2) | DRB1*14:02 (4) | DR-1402 (4) |
|  | DRB1*14:04 (1) | DR-1404 (1) | DRB1*14:04 (4) | DR-1404 (4) |
|  | DRB1*14:05 (1) | DR-1405 (1) |  |  |
| DRB1*15 | DRB1*15:01 (19) | DR-1501 (33) | DRB1*15:01 (8) | DR-1501 (32) |
|  | DRB1*15:03 (14) |  | DRB1*15:03 (24) |  |
| DRB1*16 | DRB1*16:01 (2) | DR-1601 (2) | DRB1*16:01 (4) | DR-1601 (4) |
|  | DRB1*16:02 (11) | DR-1602 (11) | DRB1*16:02 (10) | DR-1602 (10) |
